# Effect of subscapularis repair on joint contact forces based on degree of posterior-superior rotator cuff tear severity in reverse shoulder arthroplasty

**DOI:** 10.3389/fbioe.2023.1229646

**Published:** 2023-12-07

**Authors:** Donghwan Lee, Jinkyu Lee, Joo Han Oh, Choongsoo S. Shin

**Affiliations:** ^1^ Department of Mechanical Engineering, Sogang University, Seoul, Republic of Korea; ^2^ Department of Rehabilitation Medicine, Seoul National University Hospital, Seoul, Republic of Korea; ^3^ Department of Orthopaedic Surgery, Seoul National University Bundang Hospital, Seongnam, Republic Korea

**Keywords:** biomechanics, glenohumeral joint stability, musculoskeletal model, posterior-superior rotator cuff tear, reverse shoulder arthroplasty, subscapularis repair

## Abstract

Massive irreparable rotator cuff tears (RCTs) affect the clinical outcomes of reverse shoulder arthroplasty (RSA). However, the effects of subscapularis repair on the outcomes of RSA, based on the degree of posterior-superior RCTs, are unclear. This study aimed to examine the effect of subscapularis repair on three-dimensional joint contact forces (JCFs) based on the degree of posterior-superior RCT severity in RSA. Ten human *in vivo* experimental data were used as input to the musculoskeletal model. A six-degrees-of-freedom (DOF) anatomical shoulder model was developed and validated against three-dimensional JCFs. The 6-DOF musculoskeletal shoulder model of RSA was then developed by importing the reverse shoulder implant into the validated anatomical shoulder model. Based on the various types of posterior-superior RCT severity, inverse dynamic simulations of subscapularis-torn and subscapularis-repaired models of RSA were performed: from isolated supraspinatus tears to partial or massive tears of the infraspinatus and teres minor. The intact rotator cuff model of RSA was also simulated for comparison with the different types of models. Our results showed that the more posterior-superior RCTs progressed in RSA, the more superior JCFs were observed at 90°, 105°, and 120° abduction in the subscapularis-torn model. However, subscapularis repair decreased the superior JCF at those angles sufficiently. In addition, the teres minor muscle-tendon force increased as infraspinatus bundle tears progressed in both the subscapularis-torn and -repaired models, in order to compensate for the reduced force during abduction. However, the teres minor muscle-tendon force was not as high as that of the infraspinatus muscle-tendon, which could result in muscle force imbalance between repaired subscapularis and teres minor. Therefore, our results suggest that repairing the subscapularis and the repairable infraspinatus during RSA can improve glenohumeral joint stability in the superior-inferior direction by restoring muscle force balance between the anterior cuff (i.e., subscapularis) and posterior cuff (i.e., infraspinatus and teres minor). The findings of this study can help clinician decide whether to repair the rotator cuff during RSA to enhance joint stability.

## 1 Introduction

Reverse shoulder arthroplasty (RSA) is a surgical procedure used to treat pain and provide functional improvement in patients with end-stage rotator cuff tear (RCT) arthropathy (Boileau et al., 2006), pseudoparalysis with massive irreparable RCTs (Hartzler et al., 2015), trauma with fractured proximal humerus (Iacobellis et al., 2015), tumor resection (De Wilde et al., 2005; De Wilde et al., 2011), and revision arthroplasty in a rotator cuff deficient shoulder (Boileau et al., 2006; Saltzman et al., 2014). The reverse shoulder prosthetic design medializes the glenohumeral joint center of rotation, stabilizes the glenohumeral joint, and helps in recruiting more fibers of the anterior and posterior deltoid to act as abductors, thus allowing them to compensate for the deficient rotator cuff (Boileau et al., 2005). However, massive irreparable RCTs may increase the risk of glenohumeral dislocation or early loosening of the glenoid-side component (Boileau et al., 2005; Wall et al., 2007; Edwards et al., 2009). For these reasons, subscapularis repair during RSA is being considered to improve glenohumeral joint stability (Oh et al., 2014).

Various clinical outcomes have been reported following subscapularis repair in RSA. Previous studies reported that subscapularis repair can reduce dislocation rates and increase glenohumeral joint stability (Trappey et al., 2011; Matthewson et al., 2019). However, other studies reported no significant effect on dislocation rates, regardless of whether the subscapularis was repaired (Clark et al., 2012; De Fine et al., 2022). Although previous studies investigated clinical outcomes of RSA, the severity of posterior-superior RCT in preoperative conditions was not provided (Trappey et al., 2011; Clark et al., 2012; Matthewson et al., 2019; De Fine et al., 2022). Thus, how posterior-superior RCT conditions affect the outcomes of subscapularis repair in RSA is unknown.

RCT severity in preoperative patients for RSA varies from partial-to full-thickness tears of the rotator cuff (Boileau et al., 2006). A previous study reported that loss of the supraspinatus and infraspinatus reduced the joint compressive force and the glenohumeral joint stability during abduction (Ackland et al., 2019). Moreover, the absence of the rotator cuff in RSA resulted in a higher superior joint shear force, which can potentially lead to prosthesis loosening (Ackland et al., 2011). However, the effects of subscapularis repair in RSA on the joint contact forces (JCFs) based on the degree of posterior-superior RCTs are unknown. Therefore, this study aimed to examine the effect of subscapularis repair on three-dimensional JCFs based on the degree of posterior-superior RCT severity in RSA.

## 2 Methods

**FIGURE 1:**
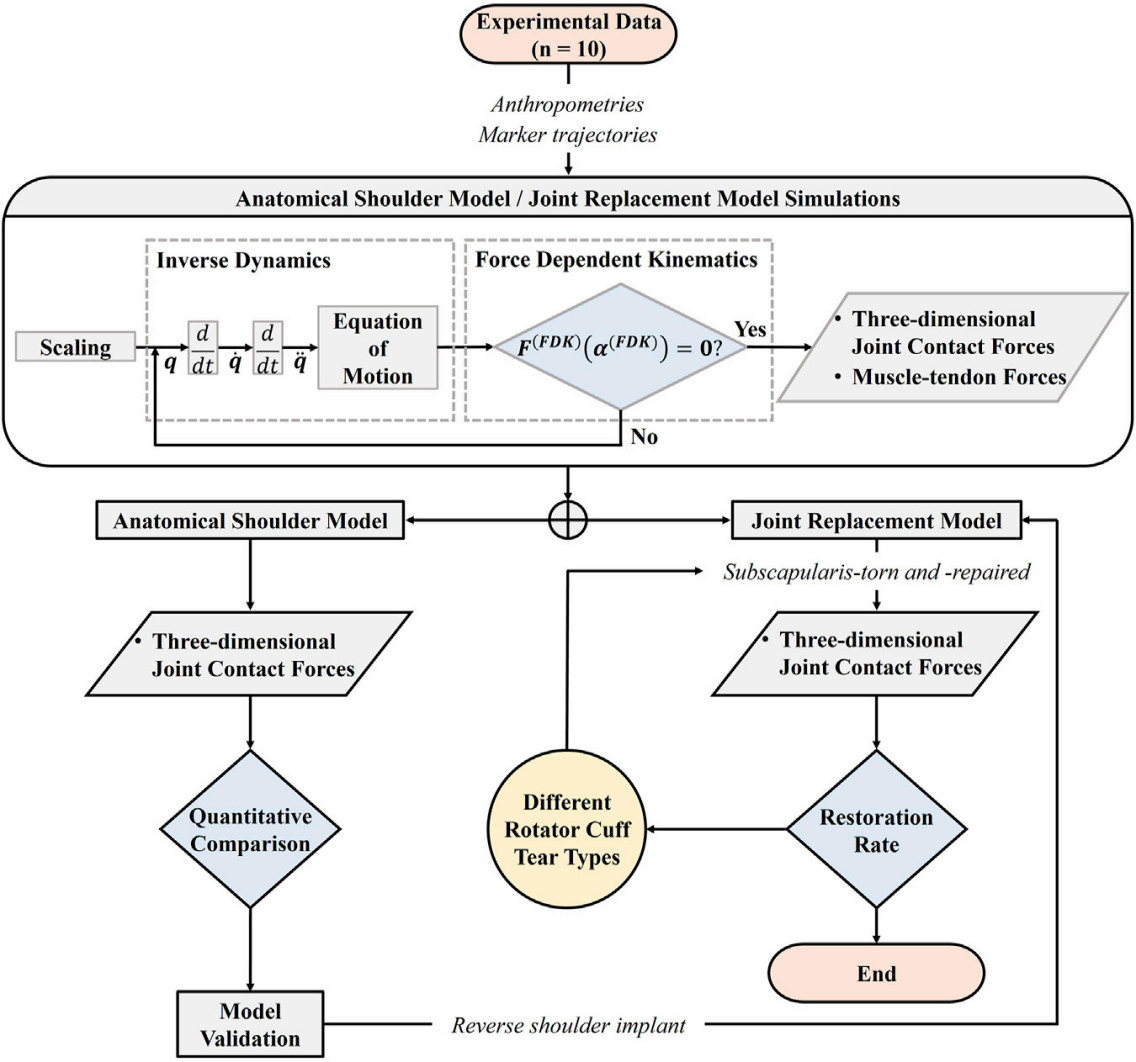
The workflow of the overall musculoskeletal modeling and simulation process using the force-dependent kinematics (FDK) method. q : joint angle; $\dot{q}$ : angular velocity; $\ddot{q}$ : angular acceleration; $F^{\left(FDK\right)}$ : FDK residual force; $\alpha^{\left(FDK\right)}$ : FDK translations at the glenohumeral joint.

The overall musculoskeletal modeling and simulation process is outlined in the workflow (Figure 1). First, ten human *in vivo* experimental data were used as input to the musculoskeletal model. Second, scaling was performed through parameter optimization to minimize the difference between model markers and experimentally recorded markers (Andersen et al., 2010). Third, inverse kinematics was performed based on the marker trajectory data, in order to calculate the positions, velocities, and accelerations of each segment; subsequently, inverse dynamics was performed. Fourth, the six-degrees-of-freedom (DOF) anatomical shoulder model was developed by allowing the medial-lateral (ML), superior-inferior (SI), and anterior-posterior (AP) translations at the glenohumeral joint using the force-dependent kinematics (FDK) method (Andersen et al., 2011). The main assumption of this method is the quasi-static equilibrium between the FDK residual force and joint translation in the FDK direction. Thus, joint translation is determined when the FDK residual force is zero for each step. Fifth, the 6-DOF anatomical shoulder model was validated against three-dimensional JCFs. Sixth, the 6-DOF musculoskeletal shoulder model of RSA was developed by importing the reverse shoulder implant into the validated anatomical shoulder model. Lastly, three-dimensional JCFs of each posterior-superior RCT severity type of subscapularis-torn and -repaired models of RSA were compared with those of the intact rotator cuff model of RSA to quantify the JCFs restoration rate.

### 2.1 Experimental protocol

**FIGURE 2:**
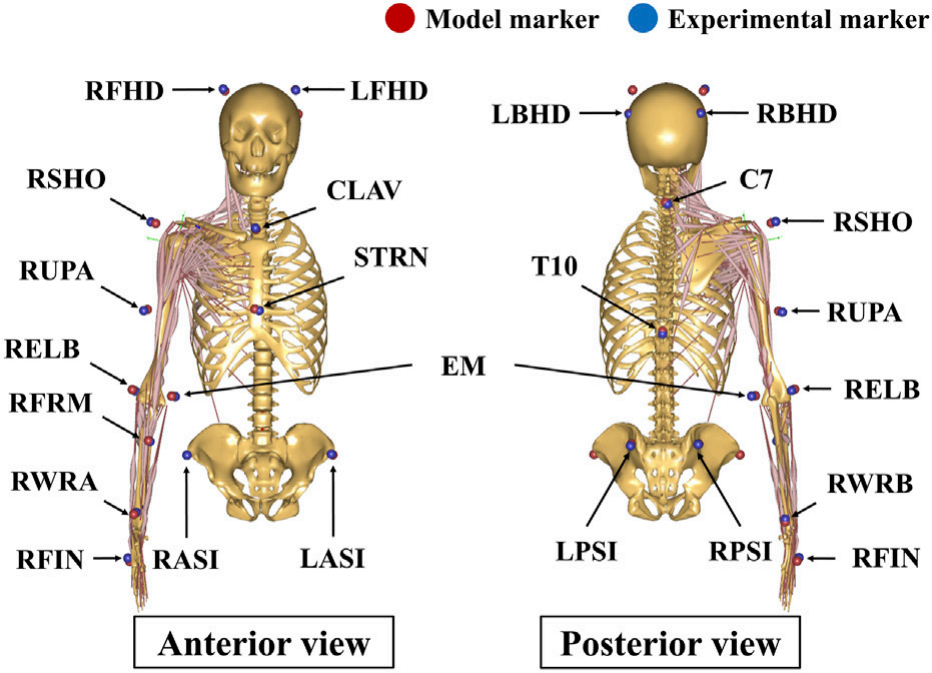
Marker placement of the upper body Plug-in-Gait model. An additional maker was attached to the medial epicondyle of the humerus based on the ISB recommendation. RFHD: right forehead; LFHD: left forehead; RBHD: right back of head; LBHD: left back of head; RSHO: right shoulder; RUPA: right upper arm; RELB: right elbow; EM: the most caudal point on medial epicondyle; RFRM: right forearm; RWRA: right wrist marker A (ulnar styloid); RWRB: right wrist marker B (radial styloid); RFIN: right finger; CLAV: clavicle; STRN: sternum; RASI: right anterior-superior iliac; LASI: left anterior-superior iliac; RPSI: right posterior-superior iliac; LPSI: left posterior-superior iliac; C7: seventh cervical vertebra; T10: 10th thoracic vertebra.

Ten male participants with no previous upper extremity injuries (age: 24.3 ± 2.1 years, weight: 75.7 ± 7.4 kg, height: 1.76 ± 0.06 m) participated in the experiments after signing an informed consent document approved by the Institutional Review Board of Sogang University. The average range of motion in abduction was less than 120° for patients undergoing RSA (Kontaxis et al., 2017). Thus, a task involving approximately 120° abduction was conducted during the experiment. Abduction took place in the coronal plane for 3 s while the elbow was fully extended with the palm facing down, returning to the resting position for 3 s, then resting for 30 s (Wu et al., 2016). Participants were instructed to elevate and return the right arm at a speed of 40°/s. Upper limb dominance was determined by asking which arm they used to write or throw a ball, and all of the participants indicated the right arm as their dominant arm. A three-dimensional motion capture system equipped with ten infrared cameras (9 Eagle, 1 Raptor; Motion Analysis Corp., Santa Rosa, CA) was used to record the motion of the glenohumeral joint at a sampling rate of 400 Hz. Retro-reflective markers were attached to the pelvis, thorax, clavicle, head, scapula, humerus, and the middle knuckle on the right hand according to the Plug-in-Gait marker set (Figure 2). An additional marker was attached to the most caudal point on the medial epicondyle of the humerus based on the International Society of Biomechanics (ISB) (Wu et al., 2005) (Figure 2). The measured kinematic data were filtered using a zero-lag fourth-order low-pass Butterworth filter at a cutoff frequency of 10 Hz.

### 2.2 Musculoskeletal model

A three-dimensional musculoskeletal generic shoulder model was implemented using the AnyBody Modeling System (V7.3.4., AnyBody Tech., Aalborg, Denmark). The deltoid group in the generic shoulder model was modeled with 12 muscle-tendon units, where each of the anterior, middle, and posterior deltoids was associated with 4 of these units. A rotator cuff group in the generic shoulder model was established, which was composed of 24 muscle-tendon units, where each of the subscapularis, supraspinatus, infraspinatus, and teres minor was associated with six of these units. Muscle forces of the generic shoulder model were estimated using the quadratic polynomial muscle recruitment criterion (Damsgaard et al., 2006). The glenohumeral joint in the generic shoulder model was modeled as a ball-and-socket joint allowing for only three-rotational DOFs. In this study, a new 6-DOF anatomical shoulder model was developed using the FDK method (Andersen et al., 2011). A linear spring element with superior glenohumeral ligament stiffness was established in this model to represent the restriction of the joint capsule and ligaments surrounding the glenohumeral joint (Boardman et al., 1996). To validate the developed anatomical shoulder model, the JCFs in the current model were compared with those in the public OrthoLoad experimental data (Bergmann et al., 2011) and a previously validated 6-DOF anatomical shoulder model (Quental et al., 2016).

**FIGURE 3:**
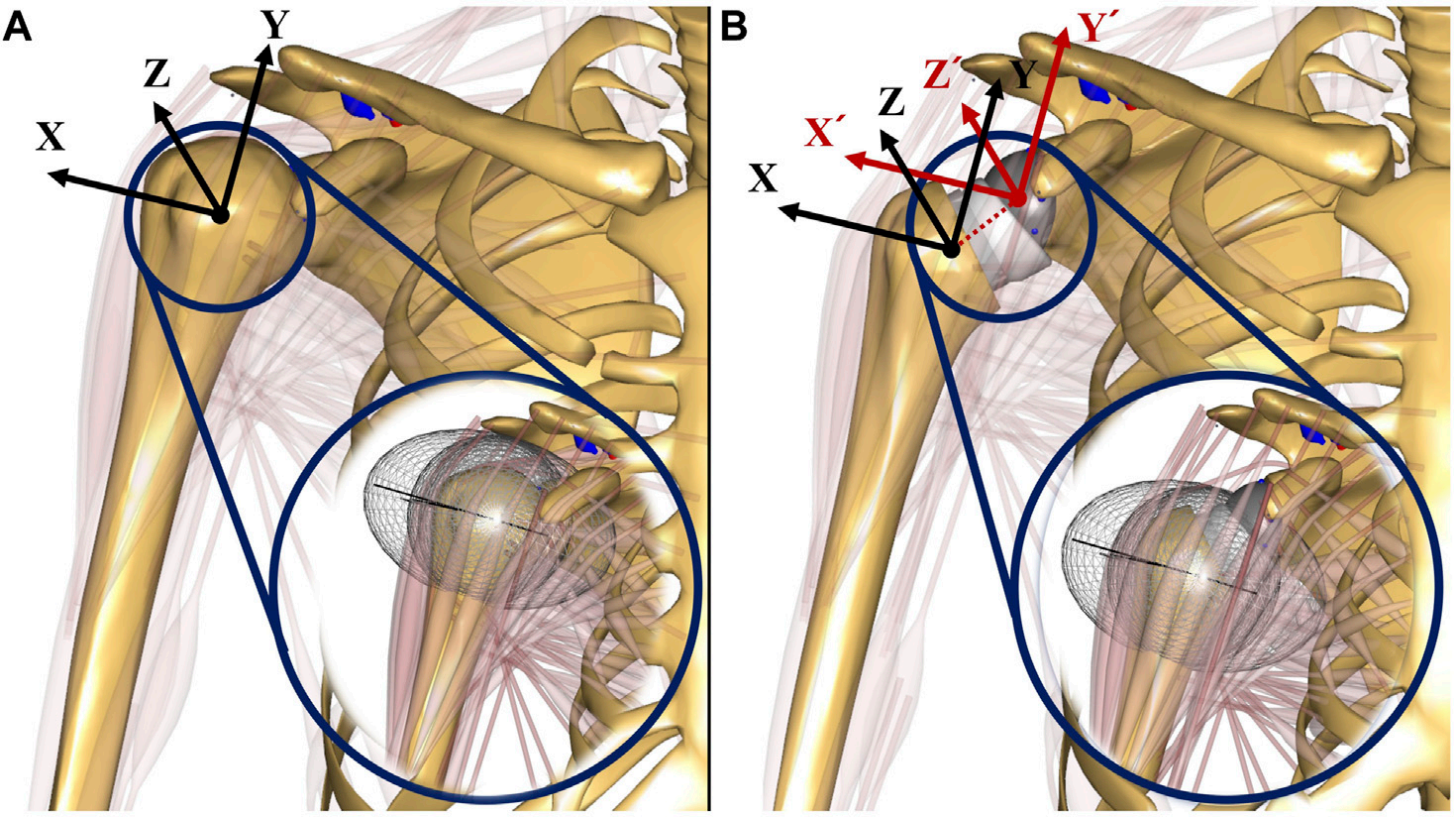
The illustrations of musculoskeletal shoulder models with the scapular reference frame: **(A)** anatomical shoulder model; **(B)** musculoskeletal model of reverse shoulder arthroplasty (RSA). The scapular reference frame in the musculoskeletal model of RSA was positioned at the rotational center of the glenosphere. X-axis, Y-axis, and Z-axis are medial-lateral, superior-inferior, and anterior-posterior, respectively.

The standard surgical guidelines for the Equinoxe Reverse Shoulder (Exactech, Gainesville, FL) were used to virtually implant the reverse shoulder prosthesis using SolidWorks 2021 (SolidWorks Corp., Concord, MA). The humeral head was resected in 20° retroversion and at a 132.5° neck angle in accordance with surgical guidelines. The reverse shoulder prosthesis exhibited an onlay design with a 132.5° neck-shaft angle of the humeral stem. A standard 38 mm non-lateralized glenosphere and an oval baseplate (33.8 mm long and 25.4 mm wide) were implanted into the scaled-generic glenoid based on surgical guidelines. Subsequently, a musculoskeletal model of RSA was developed by importing prosthetic bone geometries into a validated anatomical shoulder model. The local coordinates of the prosthetic glenohumeral joint were established by shifting the rotational center of the glenosphere from the original coordinates of the glenohumeral joint based on the scapular reference frame in the 6-DOF anatomical shoulder model (Figure 3). Changes in muscle paths after RSA were determined based on the obstacle set method (Garner and Pandy, 2000), in which an optimization algorithm was used to determine the shortest path between the origin and the insertion location of the muscle-tendon unit without penetrating several obstacles. Specifically, the location of ellipsoidal wrapping objects was shifted from a neutral position to the rotational center of the implanted humeral head; the muscle lines from origin to insertion followed the shortest path on the surface of ellipsoidal wrapping objects (Garner and Pandy, 2000) (Figure 3).

The JCFs between the glenosphere and polyethylene insert were calculated using a linear force-penetration volume law based on a contact pressure module as follows:

$$F_{i}=P_{V}∙V_{i}$$ (1)

$$P_{V}=\frac{F_{i}}{V_{i}}=\frac{p_{i}A_{i}}{A_{i}d_{i}}=\frac{\left(1-v\right)}{\left(1+v\right)\left(1-2v\right)h}\frac{2p_{0}}{\varepsilon_{0}\left[1+n{\left(\frac{p_{i}}{p_{0}}\right)}^{n-1}\right]}$$ (2)

Where $F_{i}$ is the joint contact forces at the *i*th vertex; $P_{V}$ is the contact pressure module; $V_{i}$ is the penetration volume; $p_{i}$ is the contact pressure; $A_{i}$ is the contact area; $d_{i}$ is the penetration depth; v is Poisson’s ratio of polyethylene insert; h is the thickness of polyethylene insert; Non-linear polyethylene material parameters of $\varepsilon_{0}$ = 0.0597, $p_{0}$ = 18.4 MPa, and *n* = 3 derived in a previous experimental study were used in this study (Fregly et al., 2003). Eq. 1 represents the calculation of the joint contact forces in the default FDK computational framework of the AnyBody Modeling System (Chen et al., 2014), while Eq. (2) represents the calculation of the pressure module based on elastic foundation theory (Bei and Fregly, 2004). According to Eq. 2, the pressure module was calculated as 5.03e11 N/m^3^ in this study.

### 2.3 Simulations

**FIGURE 4:**
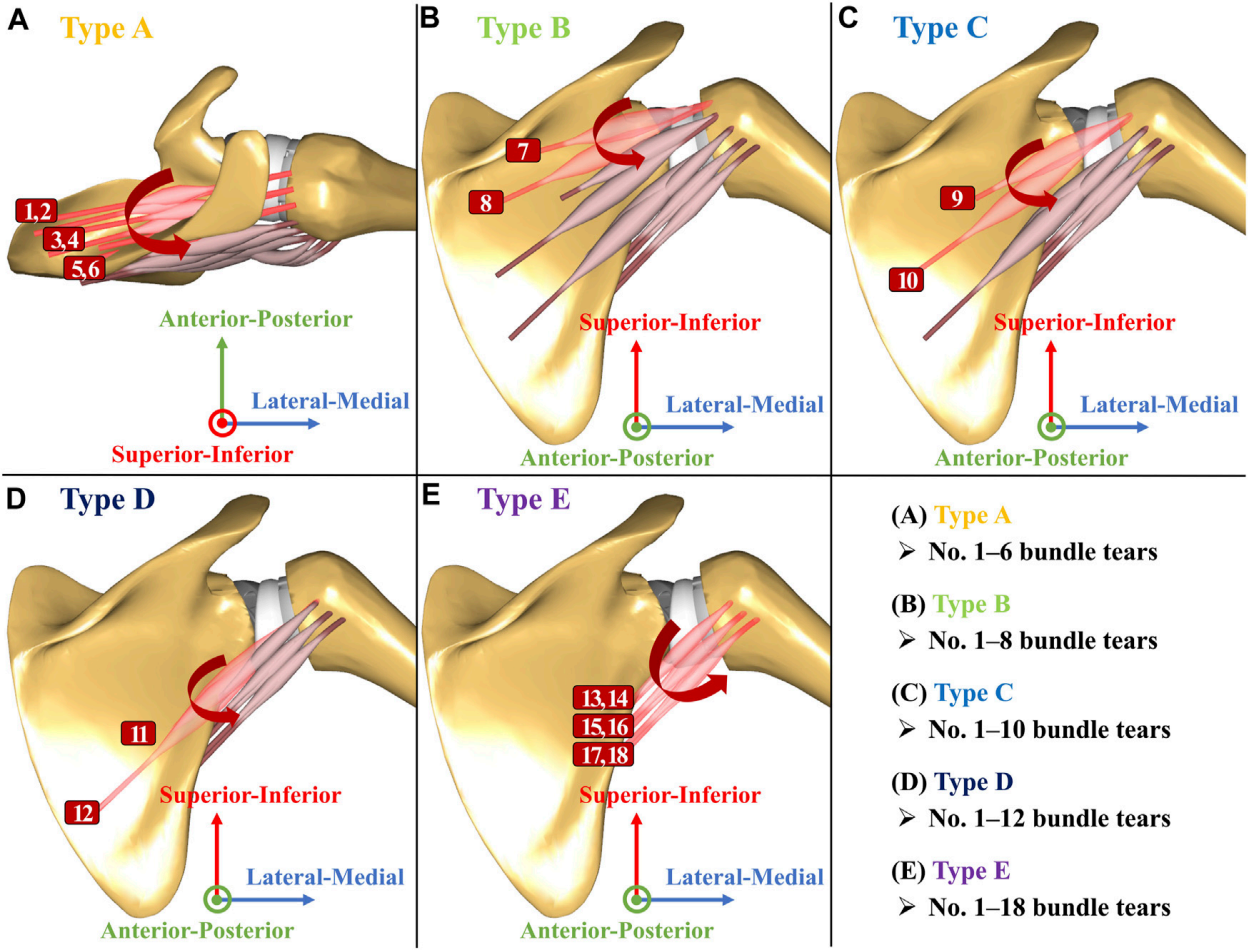
Rotator cuff configuration in the musculoskeletal model of reverse shoulder arthroplasty. **(A)** Type A: torn all six bundles of the supraspinatus (No. 1–6 bundle tears); **(B)** Type B: torn all six bundles of the supraspinatus and torn two bundles of the infraspinatus (No. 1–8 bundle tears); **(C)** Type C: torn all six bundles of the supraspinatus and torn four bundles of the infraspinatus (No. 1–10 bundle tears); **(D)** Type D: torn all six bundles of the supraspinatus and infraspinatus (No. 1–12 bundle tears); **(E)** Type E: torn all six bundles of the supraspinatus, infraspinatus, and teres minor (No. 1–18 bundle tears).

**FIGURE 5:**
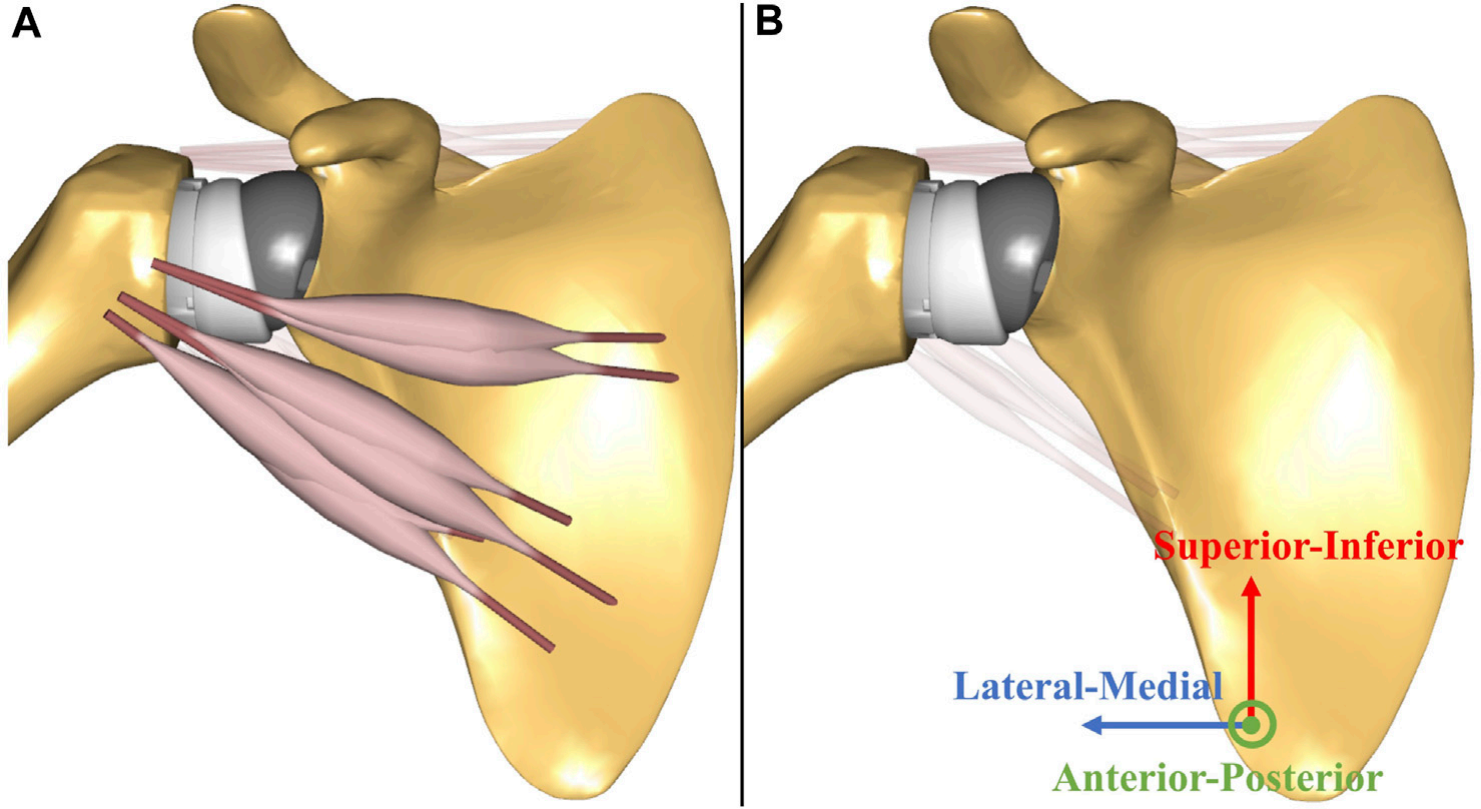
The illustrations of the subscapularis-torn and subscapularis-repaired models: **(A)** subscapularis-torn (all six bundles are torn) model; **(B)** subscapularis-repaired (all six bundles are intact) model.

RCTs from the supraspinatus extending posteriorly into the infraspinatus occur more frequently than those from the supraspinatus extending anteriorly into the subscapularis (Gerber et al., 2000; Cofield et al., 2001). The posterior-superior RCTs progressed gradually from isolated supraspinatus tears to partial or massive tears of the infraspinatus and teres minor (Kim et al., 2010; Melis et al., 2011). Thus, five types of models were established based on the degree of posterior-superior RCT severity, as the senior surgeon (JHO) suggested (Figure 4): Type A, all six bundles of the supraspinatus were torn (Figure 4A); Type B, all six bundles of the supraspinatus and two bundles (four bundles were intact) of the infraspinatus were torn (Figure 4B); Type C, six bundles of the supraspinatus and four bundles (two bundles were intact) of the infraspinatus were torn (Figure 4C); Type D, all six bundles of the supraspinatus and infraspinatus were torn (Figure 4D); and Type E, all six bundles of the supraspinatus, infraspinatus, and teres minor were torn (Figure 4E). The subscapularis-torn (all six bundles were torn) and subscapularis-repaired (all six bundles were intact) models were simulated according to the five posterior-superior RCT severity types (Figure 5). The intact rotator cuff model of RSA was also simulated to compare the JCFs with the subscapularis-torn and -repaired models.

### 2.4 Statistical analysis

The normalized JCFs (% body weight (BW)) in the ML, SI, and AP directions of the 6-DOF anatomical shoulder model were compared with previously reported JCFs (% BW) from Quental et al. (2016). The total contact force (TCF), which is the sum of the square roots of the ML-, SI-, and AP-JCF, was compared with the reported TCF. Quantitative assessments of the 6-DOF anatomical shoulder model were performed by evaluating the root-mean-squared error (RMSE) and Pearson’s correlation coefficient (*r*) compared to the human *in vivo* JCFs from 10° to 90° abduction (Bergmann et al., 2011) and compared to previously predicted JCFs from 13° to 109° abduction (Quental et al., 2016). Because of the lack of previously reported RMSEs and correlation coefficients corresponding to the intact glenohumeral JCFs, the calculated RMSEs and correlation coefficients were indirectly evaluated by comparing them with the previously reported values in the 6-DOF musculoskeletal model of total joint arthroplasty (Chen et al., 2021; Ro et al., 2022).

Post hoc paired t-tests with false discovery rate (FDR) correction were performed to compare the JCFs (% BW) and muscle-tendon forces (% BW) at 15°, 30°, 45°, 60°, 75°, 90°, 105°, and 120° abduction between the intact rotator cuff and subscapularis-torn models, and between the intact rotator cuff and subscapularis-repaired models. The Benjamini–Hochberg procedure was selected to control the FDR to 5% (Benjamini and Hochberg, 1995). All statistical analyses were performed using the MATLAB R2020b (Mathworks, Inc., Natick, MA). The JCF restoration rate of subscapularis repair was defined as the number of JCF restoration cases divided by the number of all cases (eight cases). Each case of restoration, non-restoration, and not applicable were classified as shown in Table 1.

**TABLE 1:** The description of the comparison in the joint contact forces between the intact rotator cuff and subscapularis-torn model and between the intact rotator cuff and subscapularis-repaired model.

Classification	Post hoc paired t-test results
Restoration	$F_{Intact}$ vs. $F_{SSC-torn}$ (*p < 0.05*)
	& $F_{Intact}$ vs. $F_{SSC-repaired}$ (*p > 0.05*)
Non-Restoration	$F_{Intact}$ vs. $F_{SSC-torn}$ (*p < 0.05*)
	& $F_{Intact}$ vs. $F_{SSC-repaired}$ (*p < 0.05*)
Not Applicable	$F_{Intact}$ vs. $F_{SSC-torn}$ (*p > 0.05*)

$F_{Intact}$ , normalized joint contact force of the intact rotator cuff model; $F_{SSC-torn}$ , normalized joint contact force of the subscapularis-torn model; $F_{SSC-repaired}$ , normalized joint contact force of the subscapularis-repaired model.

Restoration = There was a significant difference between $F_{Intact}$ and $F_{SSC-torn}$ . After subscapularis was repaired, there was no significant difference between $F_{Intact}$ and $F_{SSC-repaired}$ .

Non-Restoration = There was significant difference between $F_{Intact}$ and $F_{SSC-torn}$ . Although subscapularis was repaired, there was still significant difference between $F_{Intact}$ and $F_{SSC-repaired}$ .

Not Applicable = There were no significant differences between $F_{Intact}$ and $F_{SSC-torn}$ .

## 3 Results

**FIGURE 6:**
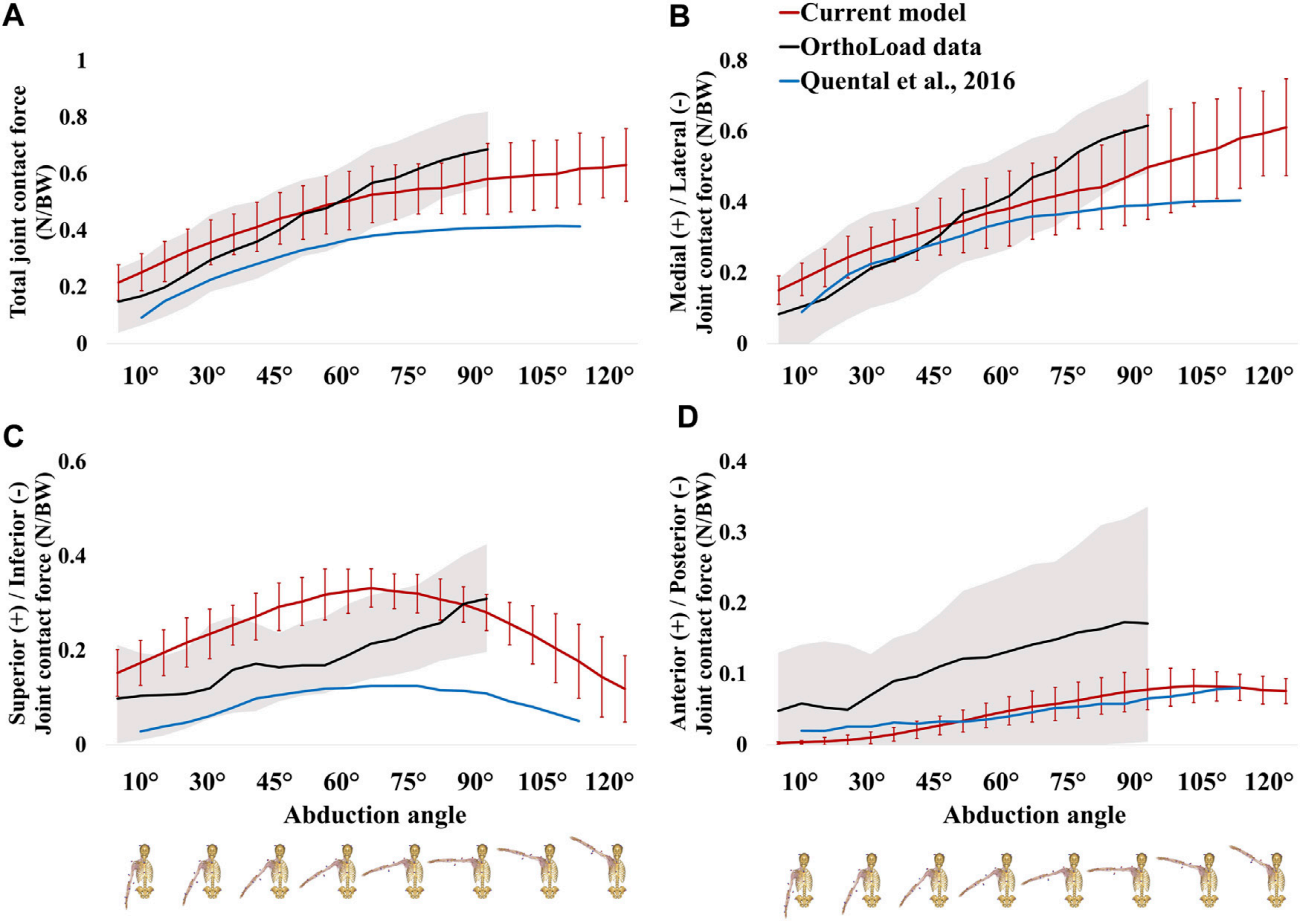
Comparison of the joint contact forces in the current model (six-degree-of-freedom (DOF) anatomical shoulder model), the public OrthoLoad experimental data, and previously validated 6-DOF anatomical shoulder model: **(A)** total joint contact force; **(B)** medial-lateral joint contact force; **(C)** superior-inferior joint contact force; **(D)** anterior-posterior joint contact force, respectively.

The magnitude and pattern of the JCFs calculated using the current (6-DOF anatomical shoulder) model are consistent with those of previously reported JCFs (Figure 6). The RMSEs of the ML-, SI-, AP-JCF, and TCF magnitudes (% BW) between the current model and human *in vivo* data were 7%, 9%, 8%, and 6%, respectively, and between the current model and previously validated 6-DOF anatomical shoulder model were 7%, 18%, 1%, and 15%, respectively. The correlation coefficients (*r*) of the ML-, SI-, AP-JCF, and TCF magnitudes (% BW) between the current model and human *in vivo* data were 0.990, 0.740, 0.983, and 0.988, respectively, and those between the current model and previously validated 6-DOF anatomical shoulder model were 0.945, 0.970, 0.964, and 0.986, respectively.

In Type A condition, significant differences were observed in the TCF between the intact rotator cuff and subscapularis-torn models at 15°, 30°, 45°, 60°, 75°, 90°, 105°, and 120° abduction. However, subscapularis repair eliminated the significant differences at 60°, 75°, 90°, 105°, and 120° abduction, resulting in a 62.5% (five among eight abduction angles) TCF restoration rate (Table 2). In Types B, C, and D conditions, the TCF restoration rate of subscapularis repair was 87.5% (Table 2); but it was 12.5% in Type E condition (Table 2).

**TABLE 2:** The effect of subscapularis repair on the restoration rate of the total joint contact force in comparison with 3 models.

Type of tears	Classification	Observed angle (degree)	Number of classifications	Restoration rate (%)
Type A	Restoration^a^	60°/75°/90°/105°/120°	5	62.5
	Non-restoration^a^ ^,^ ^b^	15°/30°/45°	3	
	Not applicable	-	0	
Type B	Restoration^a^	30°/45°/60°/75°/90°/105°/120°	7	87.5
	Non-restoration^a^ ^,^ ^b^	-	0	
	Not applicable	15°	1	
Type C	Restoration^a^	30°/45°/60°/75°/90°/105°/120°	7	87.5
	Non-restoration^a^ ^,^ ^b^	-	0	
	Not applicable	15°	1	
Type D	Restoration^a^	30°/45°/60°/75°/90°/105°/120°	7	87.5
	Non-restoration^a^ ^,^ ^b^	-	0	
	Not applicable	15°	1	
Type E	Restoration^a^	105°	1	12.5
	Non-restoration^a^ ^,^ ^b^	15°/30°/45°/60°/75°/120°	6	
	Not applicable	90°	1	

Type A: isolated bundle tear of the supraspinatus; Type B: Type A + superior bundle tear of the infraspinatus; Type C: Type B + middle bundle tear of the infraspinatus; Type D: Type C + entire bundle tear of the infraspinatus; Type E: Type D + entire bundle tear of the teres minor.

^a^ Significant differences between intact rotator cuff and subscapularis-torn models (*p* < .05).

^b^ Significant differences between intact rotator cuff and subscapularis-repaired models (*p* < .05).

In Type A condition, significant differences were observed in the joint compressive force (ML-JCF) between the intact rotator cuff and subscapularis-torn models at 15°, 30°, 45°, 60°, 75°, 90°, and 105° abduction. However, subscapularis repair eliminated the significant differences at 60°, 75°, 90°, and 105° abduction, resulting in 50% joint compressive force restoration rate (Table 3). In Types B and C conditions, the joint compressive force restoration rate of subscapularis repair was 62.5% (Table 3). In Type D condition, the joint compressive force restoration rate of subscapularis repair was 75% (Table 3); but it was 0% in Type E condition (Table 3).

**TABLE 3:** The effect of subscapularis repair on the restoration rate of the joint compressive force (medial-lateral joint contact force) in comparison with 3 models.

Type of tears	Classification	Observed angle (degree)	Number of classifications	Restoration rate (%)
Type A	Restoration^a^	60°/75°/90°/105°	4	50
	Non-restoration^a^ ^,^ ^b^	15°/30°/45°	3	
	Not applicable	120°	1	
Type B	Restoration^a^	45°/60°/75°/90°/105°	5	62.5
	Non-restoration^a^ ^,^ ^b^	15°/30°	2	
	Not applicable	120°	1	
Type C	Restoration^a^	45°/60°/75°/90°/105°	5	62.5
	Non-restoration^a^ ^,^ ^b^	15°/30°	2	
	Not applicable	120°	1	
Type D	Restoration^a^	45°/60°/75°/90°/105°/120°	6	75
	Non-restoration^a^ ^,^ ^b^	15°/30°	2	
	Not applicable	-	0	
Type E	Restoration^a^	-	0	0
	Non-restoration^a^ ^,^ ^b^	15°/30°/45°/60°/75°/105°/120°	7	
	Not applicable	90°	1	

Type A: isolated bundle tear of the supraspinatus; Type B: Type A + superior bundle tear of the infraspinatus; Type C: Type B + middle bundle tear of the infraspinatus; Type D: Type C + entire bundle tear of the infraspinatus; Type E: Type D + entire bundle tear of the teres minor.

^a^ Significant differences between intact rotator cuff and subscapularis-torn models (*p* < .05).

^b^ Significant differences between intact rotator cuff and subscapularis-repaired models (*p* < .05).

In Types A and C conditions, significant differences were observed in the SI-JCF between the intact rotator cuff and subscapularis-torn models at 15°, 30°, 45°, 60°, 75°, 90°, 105°, and 120° abduction. However, subscapularis repair eliminated the significant differences at 75°, 90°, 105°, and 120° abduction, resulting in 50% SI-JCF restoration rate (Table 4). In Type B condition, the SI-JCF restoration rate of subscapularis repair was 62.5% (Table 4); but it was less than 50% in Types D and E conditions (Table 4).

**TABLE 4:** The effect of subscapularis repair on the restoration rate of the superior-inferior joint contact force in comparison with 3 models.

Type of tears	Classification	Observed angle (degree)	Number of classifications	Restoration rate (%)
Type A	Restoration^a^	75°/90°/105°/120°	4	50
	Non-restoration^a^ ^,^ ^b^	15°/30°/45°/60°	4	
	Not applicable	-	0	
Type B	Restoration^a^	60°/75°/90°/105°/120°	5	62.5
	Non-restoration^a^ ^,^ ^b^	15°/30°/45°	3	
	Not applicable	-	0	
Type C	Restoration^a^	75°/90°/105°/120°	4	50
	Non-restoration^a^ ^,^ ^b^	15°/30°/45°/60°	4	
	Not applicable	-	0	
Type D	Restoration^a^	90°/105°/120°	3	37.5
	Non-restoration^a^ ^,^ ^b^	15°/30°/45°/60°/75°	5	
	Not applicable	-	0	
Type E	Restoration^a^	120°	1	12.5
	Non-restoration^a^ ^,^ ^b^	15°/30°/45°/60°	4	
	Not applicable	75°/90°/105°	3	

Type A: isolated bundle tear of the supraspinatus; Type B: Type A + superior bundle tear of the infraspinatus; Type C: Type B + middle bundle tear of the infraspinatus; Type D: Type C + entire bundle tear of the infraspinatus; Type E: Type D + entire bundle tear of the teres minor.

^a^ Significant differences between intact rotator cuff and subscapularis-torn models (*p* < .05).

^b^ Significant differences between intact rotator cuff and subscapularis-repaired models (*p* < .05).

In Type A condition, significant differences were observed in the AP-JCF between the intact rotator cuff and subscapularis-torn models at 15°, 30°, 45°, 60°, 90°, 105°, and 120° abduction. However, subscapularis repair eliminated the significant differences at 90° and 105° abduction, resulting in a 25% AP-JCF restoration rate (Table 5). In Types B, C, D, and E conditions, the AP-JCF restoration rate of subscapularis repair was 0% (Table 5).

**TABLE 5:** The effect of subscapularis repair on the restoration rate of the anterior-posterior joint contact force in comparison with 3 models.

Type of tears	Classification	Observed angle (degree)	Number of classifications	Restoration rate (%)
Type A	Restoration^a^	90°/105°	2	25
	Non-restoration^a^ ^,^ ^b^	15°/30°/45°/60°/75°/120°	6	
	Not applicable	-	0	
Type B	Restoration^a^	-	0	0
	Non-restoration^a^ ^,^ ^b^	15°/30°/45°/60°/75°/90°/105°/120°	8	
	Not applicable	-	0	
Type C	Restoration^a^	-	0	0
	Non-restoration^a^ ^,^ ^b^	15°/30°/45°/60°/75°/90°/105°/120°	8	
	Not applicable	-	0	
Type D	Restoration^a^	-	0	0
	Non-restoration^a^ ^,^ ^b^	15°/30°/45°/60°/75°/90°/105°/120°	8	
	Not applicable	-	0	
Type E	Restoration^a^	-	0	0
	Non-restoration^a^ ^,^ ^b^	15°/30°/45°/60°	4	
	Not applicable	75°/90°/105°/120°	4	

Type A: isolated bundle tear of the supraspinatus; Type B: Type A + superior bundle tear of the infraspinatus; Type C: Type B + middle bundle tear of the infraspinatus; Type D: Type C + entire bundle tear of the infraspinatus; Type E: Type D + entire bundle tear of the teres minor.

^a^ Significant differences between intact rotator cuff and subscapularis-torn models (*p* < .05).

^b^ Significant differences between intact rotator cuff and subscapularis-repaired models (*p* < .05).

**FIGURE 7:**
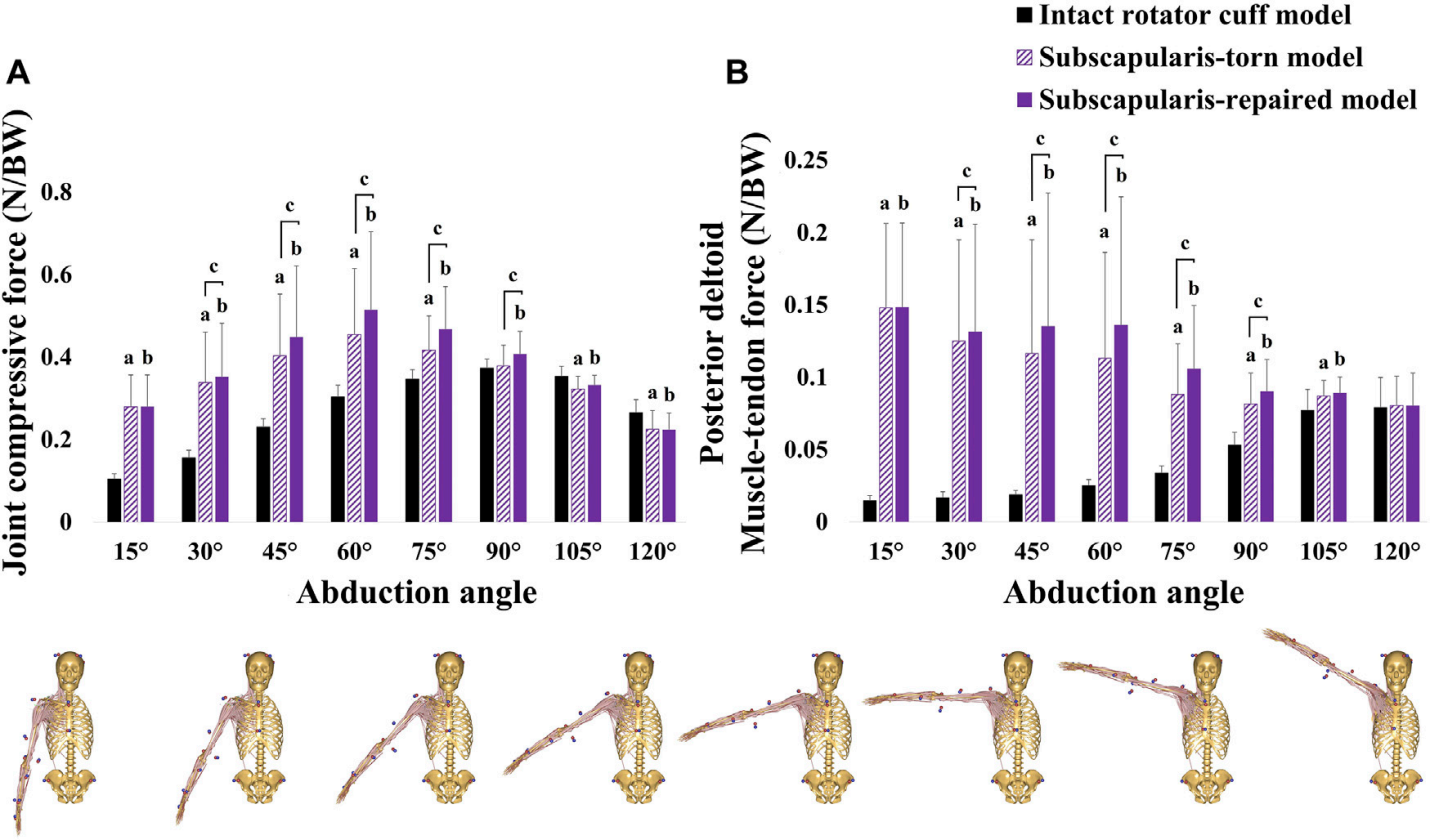
**(A)** joint compressive force (medial-lateral joint contact force) and **(B)** posterior deltoid muscle-tendon force in the Type E condition (torn all six bundles of the supraspinatus, infraspinatus, and teres minor). ^a^Significant differences between intact rotator cuff and subscapularis-torn models (*p* < .05). ^b^Significant differences between intact rotator cuff and subscapularis-repaired models (*p* < .05). ^c^Significant differences between subscapularis-torn and -repaired models (*p* < .05).

In Types A, B, C, and D conditions, each joint compressive force of both the subscapularis-torn and -repaired models was not significantly higher than that of the intact rotator cuff model; however, in Type E condition, it was significantly higher than that of the intact rotator cuff model at 15°, 30°, 45°, 60°, and 75° abduction (Figure 7A).

In Type E condition, each posterior deltoid muscle-tendon force in the subscapularis-torn and -repaired models was significantly higher than that of the intact rotator cuff model at 15°, 30°, 45°, 60°, 75°, 90°, and 105° abduction (Figure 7B).

**FIGURE 8:**
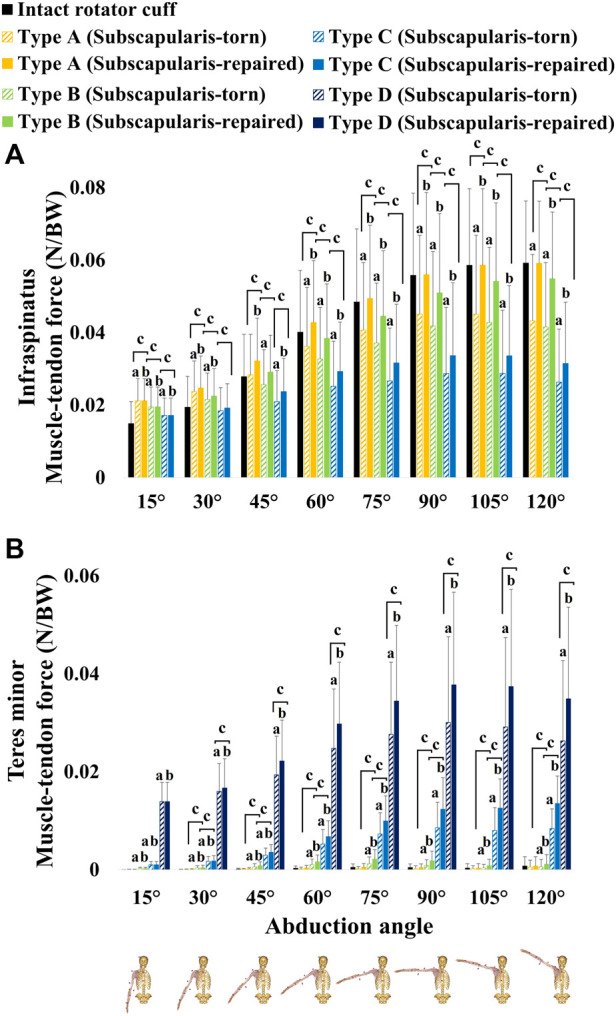
**(A)** infraspinatus muscle-tendon force and **(B)** teres minor muscle-tendon force. Type A: isolated bundle tear of the supraspinatus; Type B: Type A + superior bundle tear of the infraspinatus; Type C: Type B + middle bundle tear of the infraspinatus; Type D: Type C + entire bundle tear of the infraspinatus.

In all conditions, each infraspinatus muscle-tendon force in the subscapularis-repaired model was significantly increased at 30°, 45°, 60°, 75°, 90°, 105°, and 120° abduction than that of the subscapularis-torn model (Figure 8A).

In Type A condition, no significant differences were observed in the teres minor muscle-tendon force between the subscapularis-torn and -repaired models (Figure 8B). In Type B condition, the teres minor muscle-tendon force of the subscapularis-repaired model was significantly increased at 30°, 45°, 60°, 75°, 90°, and 120° abduction compared with that of the subscapularis-torn model (Figure 8B). In Types C and D conditions, the teres minor muscle-tendon force of the subscapularis-repaired model was significantly increased at 30°, 45°, 60°, 75°, 90°, 105°, and 120° abduction compared with that of the subscapularis-torn model (Figure 8B).

## 4 Discussion

This study is the first to use a validated anatomical shoulder model to develop a 6-DOF musculoskeletal shoulder model of RSA, allowing three translations of the glenohumeral joint. As this study demonstrated that the maximum RMSE (0.18 BW) and minimum correlation coefficient (*r* = 0.740) are within the reported range of RMSEs (0.12–0.28 BW) and correlation coefficients (0.701–0.962) of previous studies (Chen et al., 2021; Ro et al., 2022), the 6-DOF musculoskeletal shoulder model of RSA proposed herein appears to predict JCFs during abduction sufficiently.

The main finding of this study was that JCF restoration of subscapularis repair in RSA was considerably affected by posterior-superior RCT severity. Our results showed that the TCF restoration rate of subscapularis repair exceeded 50% in the tears extending posteriorly from the isolated supraspinatus into the infraspinatus (Table 2). However, the restoration rate in the entire tears of the supraspinatus, infraspinatus, and teres minor was found to be 12.5% (Table 2). These results indicate that subscapularis repair in RSA exerts a considerable restorative effect on diminished TCF when the infraspinatus or teres minor remains. Currently, the clinical outcomes of subscapularis repair in RSA are controversial. Some studies reported that this procedure can improve glenohumeral joint stability (Trappey et al., 2011; Matthewson et al., 2019), whereas others reported that it does not affect joint stability (Clark et al., 2012; De Fine et al., 2022). These studies (Trappey et al., 2011; Clark et al., 2012; Matthewson et al., 2019; De Fine et al., 2022) did not report the severity of posterior-superior RCT conditions when investigating the clinical outcomes of RSA. Based on our results on the different restoration rate, studies reported positive effects of subscapularis repair (Trappey et al., 2011; Matthewson et al., 2019) may include a relatively lower proportion of RSA patients with the entire tears of the supraspinatus, infraspinatus, and teres minor. Conversely, other studies (Clark et al., 2012; De Fine et al., 2022) may have a relatively higher proportion of RSA patients with those tears, resulting in no effect of subscapularis repair. Therefore, this study may partially explain the conflicting clinical outcomes of subscapularis repair in RSA.

The joint compressive force restoration rate of subscapularis repair exceeded 50% when the rotator cuff was torn posteriorly from the isolated supraspinatus to both the supraspinatus and infraspinatus; this occurred because the repaired subscapularis sufficiently increased the compressive force compared with the intact rotator cuff model (Table 3; Supplementary Table S1). These results are consistent with those of a previous study, which reported that the deltoid and subscapularis lines of action increased compressive force during abduction, enhancing joint stability (Ackland et al., 2011). In this study, restoration was not observed when the supraspinatus, infraspinatus, and teres minor were torn, and the compressive force increased significantly compared with that of the intact rotator cuff model (Figure 7A). This is caused by the compensated posterior deltoid muscle-tendon force for the tears of the supraspinatus, infraspinatus, and teres minor, regardless of the subscapularis repairs at 15°, 30°, 45°, 60°, and 75° abduction (Figure 7B). When the subscapularis was repaired, a significantly high posterior deltoid muscle-tendon force and joint compressive force were observed due to the co-contraction of the subscapularis and posterior deltoid (Figure 7). The muscle force balance between the anterior (i.e., subscapularis) and posterior (i.e., infraspinatus and teres minor) rotator cuff, often referred to as the transverse force couple, is a critical component of joint stability because it provides concavity compression at the glenohumeral joint (Thompson et al., 1996). The absence of the posterior rotator cuff in RSA resulted in decreased joint compressive force and can lead to glenohumeral joint instability (Ackland et al., 2011; Ackland et al., 2019). The excessive increase in the posterior deltoid force and glenohumeral joint loads can reduce the long-term life of the reverse prosthesis (Onstot et al., 2012; Hansen et al., 2015); thus, subscapularis repair may not be recommended when the supraspinatus, infraspinatus, and teres minor are torn. Cumulatively, subscapularis repair in RSA can restore joint stability in the ML direction even when a small portion of the posterior rotator cuff remains. However, not repairing the subscapularis is advantageous when the supraspinatus, infraspinatus, and teres minor are torn.

The SI-JCF restoration rate of subscapularis repair exceeded 50% in the tears extending posteriorly from the isolated supraspinatus into the superior-middle infraspinatus, but it was less than 50% when the entire infraspinatus or teres minor was torn (Table 4). Although the restoration rate was less than 50% from the entire tears of the infraspinatus, SI-JCF restoration was observed at 90°, 105°, and 120° abduction, except when the supraspinatus, infraspinatus, and teres minor were torn (Table 4). The current results demonstrated that the increased superior JCF of the subscapularis-torn model decreased sufficiently in the subscapularis-repaired model, thereby eliminating significant differences from the intact rotator cuff model at 90°, 105°, and 120° abduction (Supplementary Table S2). These results can be explained by the muscle-tendon force of the posterior rotator cuff increasing when the subscapularis was repaired. This occurred due to muscle force balance in the transverse plane between the repaired subscapularis and posterior rotator cuff (Figure 8); thus, restoration was observed at 90°, 105°, and 120° abduction. The muscle force balance between the anterior and posterior rotator cuff maintains glenohumeral joint stability in the SI direction (Thompson et al., 1996). The teres minor muscle-tendon force increased as infraspinatus bundle tears progressed in both the subscapularis-torn and -repaired models, in order to compensate for the reduced force during abduction. (Figure 8B). These results are consistent with those of a previous study (Ackland et al., 2019), which showed that the teres minor force increased during abduction when the supraspinatus and infraspinatus were torn in RSA. However, the teres minor muscle-tendon force was not generated as high as the infraspinatus muscle-tendon force in this study (Figure 8B). These outcomes could result in a muscle force imbalance with the subscapularis muscle-tendon force during abduction. Therefore, our results suggest that repairing the subscapularis and the repairable infraspinatus during RSA can improve glenohumeral joint stability in the SI direction by restoring muscle force balance between the anterior and posterior rotator cuff.

This study has some limitations. First, this study did not demonstrate the effects of other partial RCTs, such as supraspinatus repair. Although the partial- or full-thickness repair of the supraspinatus may provide functional improvement, patient satisfaction, and resolution of painful symptoms (Fama et al., 2021), most of the indications for patients undergoing RSA had a technically irreparable tear of the supraspinatus (Lacheta et al., 2020). Second, because only men were included in this study, the results may not be generalized to women. However, according to a previous study (Cigolotti et al., 2021), sex did not affect the clinical outcomes of patients who underwent subscapularis repair. Thus, the results of this study may not be affected by sex differences; but future studies are needed to evaluate whether the subscapularis repair outcomes of RSA differ by sex. Third, the subscapularis-repaired model assumes that the entire repaired subscapularis is completely attached to the lesser tuberosity of the humerus. In previous studies, which reported that subscapularis repair did not affect the clinical outcomes of RSA, the integrity of the repaired subscapularis was unknown (Vourazeris et al., 2017; Franceschetti et al., 2019). However, de Boer et al. (2016) reported that 40% of the repaired subscapularis in RSA remained at the lesser tuberosity of the humerus after a 36-month follow-up ultrasonographic examination. Additionally, Collin et al. (2022) reported that the integrity of the repaired subscapularis affected the clinical outcomes of patients undergoing RSA. Cumulatively, the muscle force imbalance due to the re-rupture of the repaired subscapularis can provide additional evidence for the conflicting outcomes of subscapularis repair in RSA. Further studies are required to determine the effect of the re-rupture rate of the repaired subscapularis on subscapularis repair. Furthermore, this study does not account for the level of surgical difficulty in posterior rotator cuff repair during RSA. Our findings may be adapted to the completion degree of posterior-superior rotator cuff repair in clinical surgery. Therefore, further research is required to evaluate the effect of subscapularis repair based on the degree of posterior-superior rotator cuff repair completion on joint biomechanics in RSA.

## 5 Conclusion

This study demonstrated that the effect of subscapularis repair on JCF restoration in RSA was affected by the degree of posterior-superior RCT severity. Additionally, our results demonstrated that subscapularis repair significantly benefitted the restoration of glenohumeral joint stability in the ML direction, even when a portion of the posterior-superior rotator cuff remained. However, the infraspinatus tear in RSA could result in glenohumeral joint instability in the SI direction due to muscle force imbalance between the teres minor and the repaired subscapularis during abduction. Therefore, repairing the subscapularis and the repairable infraspinatus during RSA can improve glenohumeral joint stability in the SI direction by restoring muscle force balance between the anterior and posterior rotator cuff. The findings of this study can help clinician decide whether to repair the rotator cuff during RSA to enhance joint stability.

## Data Availability

The datasets for this article are not publicly available due to concerns regarding participant/patient anonymity. Requests to access the datasets should be directed to the corresponding author.
