# Supplementary material for: Effect of subscapularis repair on joint contact forces based on degree of posterior-superior rotator cuff tear severity in reverse shoulder arthroplasty
Source: Front Bioeng Biotechnol. 2023 Dec 7;11:1229646. doi: 10.3389/fbioe.2023.1229646 (PMC10733495; doi:10.3389/fbioe.2023.1229646)
Supplement: Supplementary file 1 [file Table1.DOCX]

Supplementary Material

Supplementary Table S1 Differences in the joint compressive force (medial-lateral joint contact force) between the intact rotator cuff and subscapularis-torn models, and between the intact rotator cuff and subscapularis-repaired models, respectively.

| Type of tears (model) | Abduction angle | | | | | |
| --- | --- | --- | --- | --- | --- | --- |
|  | 45° | 60° | 75° | 90° | 105° | 120° |
| Type A  (SSC-torn) | -0.0129  ± 0.0038 | -0.0189  ± 0.0055 | -0.0211  ± 0.0110 | -0.0182  ± 0.0152 | -0.0139  ± 0.0163 | -0.0086  ± 0.0141 |
| Type A  (SSC-repaired) | -0.0010  ± 0.0005 | -0.0004  ± 0.0007 | -0.0002  ± 0.0005 | -0.0001  ± 0.0003 | -0.0002  ± 0.0005 | -0.0001  ± 0.0005 |
| *P*-value | < .001 | < .001 | < .001 | .0040 | .0260 | .0891 |
| Type B  (SSC-torn) | -0.0129  ± 0.0043 | -0.0187  ± 0.0058 | -0.0207  ± 0.107 | -0.0177  ± 0.0149 | -0.0137  ± 0.0161 | -0.0089  ± 0.0143 |
| Type B  (SSC-repaired) | -0.0008  ± 0.0013 | -0.0001  ± 0.0016 | 0.0004  ± 0.0014 | -0.0001  ± 0.0032 | -0.0010  ± 0.0032 | -0.0014  ± 0.0019 |
| *P*-value | .0032 | < .001 | < .001 | .0132 | .0679 | .1682 |
| Type C  (SSC-torn) | -0.0134  ± 0.0048 | -0.0193  ± 0.0055 | -0.0217  ± 0.0080 | -0.0178  ± 0.0122 | -0.0137  ± 0.0141 | -0.0097  ± 0.0133 |
| Type C  (SSC-repaired) | -0.0013  ± 0.0022 | -0.0004  ± 0.0029 | 0.0003  ± 0.0029 | 0.0004  ± 0.0054 | -0.0008  ± 0.0061 | -0.0022  ± 0.0043 |
| *P*-value | < .001 | < .001 | < .001 | .0087 | .0701 | .2022 |
| Type D  (SSC-torn) | -0.0143  ± 0.0060 | -0.0194  ± 0.0081 | -0.0224  ± 0.0079 | -0.0191  ± 0.0075 | -0.0156  ± 0.0065 | -0.0125  ± 0.0086 |
| Type D  (SSC-repaired) | -0.0022  ± 0.0049 | 0.0007  ± 0.0099 | 0.0018  ± 0.0109 | 0.0016  ± 0.0125 | -0.0009  ± 0.0096 | -0.0037  ± 0.0061 |
| *P*-value | < .001 | < .001 | < .001 | < .001 | .0139 | .0690 |
| Type E  (SSC-torn) | 0.1732  ± 0.1373 | 0.1515  ± 0.1406 | 0.0701  ± 0.0693 | 0.0058  ± 0.0344 | -0.0313  ± 0.0157 | -0.0407  ± 0.025 |
| Type E  (SSC-repaired) | 0.2182  ± 0.1613 | 0.2117  ± 0.1702 | 0.1213  ± 0.0894 | 0.0342  ± 0.0113 | -0.0212  ± 0.0113 | -0.0413  ± 0.0245 |
| *P*-value | < .001 | < .001 | < .001 | < .001 | .0727 | .0859 |

Data are presented as mean difference joint compressive force (N/BW) ± standard deviation.

SSC: subscapularis; Type A: isolated bundle tear of the supraspinatus; Type B: Type A + superior bundle tear of the infraspinatus; Type C: Type B + middle bundle tear of the infraspinatus; Type D: Type C + entire bundle tear of the infraspinatus; Type E: Type D + entire bundle tear of the teres minor.
