# Supplementary material for: Effect of subscapularis repair on joint contact forces based on degree of posterior-superior rotator cuff tear severity in reverse shoulder arthroplasty
Source: Front Bioeng Biotechnol. 2023 Dec 7;11:1229646. doi: 10.3389/fbioe.2023.1229646 (PMC10733495; doi:10.3389/fbioe.2023.1229646)
Supplement: Supplementary file 2 [file Table2.DOCX]

Supplementary Material

Supplementary Table S2 Differences in the superior-inferior joint contact force between the intact rotator cuff and subscapularis-torn models, and between the intact rotator cuff and subscapularis-repaired models, respectively.

| Type of tears (model) | Abduction angle | | | | |
| --- | --- | --- | --- | --- | --- |
|  | 60° | 75° | 90° | 105° | 120° |
| Type A  (SSC-torn) | 0.0080  ± 0.0011 | 0.0152  ± 0.0043 | 0.2430  ± 0.0079 | 0.0326 ±0.0117 | 0.0387  ± 0.0141 |
| Type A  (SSC-repaired) | 0.0006  ± 0.0003 | 0.0001  ± 0.0003 | -0.00002  ± 0.0003 | 0.00004  ± 0.0001 | -0.0004  ± 0.0010 |
| *P*-value | < .001 | < .001 | < .001 | < .001 | < .001 |
| Type B  (SSC-torn) | 0.0076  ± 0.0013 | 0.0145  ± 0.0039 | 0.0227  ± 0.0074 | 0.0309  ± 0.0110 | 0.0379  ± 0.0138 |
| Type B  (SSC-repaired) | 0.0002  ± 0.0007 | -0.0006  ± 0.0009 | -0.0004  ± 0.0039 | 0.0002  ± 0.0070 | 0.0017  ± 0.0070 |
| *P*-value | .3324 | < .001 | < .001 | < .001 | < .001 |
| Type C  (SSC-torn) | 0.0085  ± 0.0014 | 0.0158  ± 0.0031 | 0.0236  ± 0.0056 | 0.0312  ± 0.0087 | 0.0391  ± 0.0122 |
| Type C  (SSC-repaired) | 0.0013  ± 0.0010 | 0.0008  ± 0.0015 | 0.0009  ± 0.0053 | 0.0026  ± 0.0114 | 0.0058  ± 0.0145 |
| *P*-value | .0027 | < .001 | < .001 | < .001 | .0025 |
| Type D  (SSC-torn) | 0.0125  ± 0.0036 | 0.0196  ± 0.0025 | 0.0274  ± 0.0041 | 0.0355  ± 0.0023 | 0.0447  ± 0.0052 |
| Type D  (SSC-repaired) | 0.0060  ± 0.0030 | 0.0044  ± 0.0019 | 0.0036  ± 0.0089 | 0.0062  ± 0.0156 | 0.0109  ± 0.0197 |
| *P*-value | < .001 | < .001 | < .001 | < .001 | < .001 |
| Type E  (SSC-torn) | 0.0294  ± 0.0268 | -0.0130  ± 0.0362 | -0.0100  ± 0.0295 | 0.0151  ± 0.0216 | 0.0383  ± 0.0179 |
| Type E  (SSC-repaired) | 0.0243  ± 0.0310 | -0.0401  ± 0.0466 | -0.0469  ± 0.0430 | -0.0222  ± 0.0369 | 0.0016  ± 0.0327 |
| *P*-value | .0352 | < .001 | < .001 | .0542 | < .001 |

Data are presented as mean difference superior-inferior joint contact force (N/BW) ± standard deviation.

SSC: subscapularis; Type A: isolated bundle tear of the supraspinatus; Type B: Type A + superior bundle tear of the infraspinatus; Type C: Type B + middle bundle tear of the infraspinatus; Type D: Type C + entire bundle tear of the infraspinatus; Type E: Type D + entire bundle tear of the teres minor.
